# Supplementary material for: Chemopreventive effect of pomegranate and cocoa extracts on ultraviolet radiation-induced photocarcinogenesis in SKH-1 mice
Source: PLoS One. 2020 Apr 30;15(4):e0232009. doi: 10.1371/journal.pone.0232009 (PMC7192448; doi:10.1371/journal.pone.0232009)
Supplement: Attachment — Submitted filename: Answers_to_the_reviewer.pdf [file pone.0232009.s002.pdf]

We shall write the comments and suggestions of the reviewer in boldface letters and our comments to them in normal letters.

Answers to remarks from editor

To enhance the reproducibility of your results, we recommend that if applicable you deposit your laboratory protocols in protocols.io, where a protocol can be assigned its own identifier (DOI) such that it can be cited independently in the future. For instructions see: http://journals.plos.org/plosone/s/submission-guidelines#loc-laboratory-protocols

We have published our photocarcinogenesis protocol in protocols.io as "Skin photocarcinogenesis by sunbed in SKH-1 mice".

When submitting your revision, we need you to address these additional requirements. Please ensure that your manuscript meets PLOS ONE's style requirements, including those for file naming...

We have followed all instructions to prepare this revision.

As part of your revision, please complete and submit a copy of the ARRIVE Guidelines checklist, a document that aims to improve experimental reporting and reproducibility of animal studies for purposes of post-publication data analysis and reproducibility: https://www.nc3rs.org.uk/arrive-guidelines. Please include your completed checklist as a Supporting Information file. Note that if your paper is accepted for publication, this checklist will be published as part of your article.

We have included this file as Supporting Information file and we have followed all the rules stated ARRIVE Guidelines.

We noticed you have some minor occurrence(s) of overlapping text with the following previous publication(s), which needs to be addressed: https://doi.org/10.1111/jop.12435 In your revision ensure you cite all your sources (including your own works), and quote or rephrase any duplicated text outside the Methods section. Further consideration is dependent on these concerns being addressed.

The unique coincidence detected with paper https://doi.org/10.1111/jop.12435 is stated in subsection "Statistical analysis". We have rewritten it for avoiding such intersection. Paper https://doi.org/10.1111/jop.12435 has not been included at References section because it is not related with our research in the present paper.

We note that you have not given a rationale for the chosen treatment doses. Please elaborate this aspect in the Methods section. Please also justify why group 3 received "UVR" radiation, if this is a misspelling please correct it.

The doses of the agents were selected in order to provide equivalent concentrations with other anticancer researches where has been previously tested the positive effect of these compounds (Vlachojannis et al, 2015, doi: 10.1155/2015/258598) Urpi-Sarda et al 2009 (10.1007/s00216-009-2676-1). UVR is the abbreviation of ultraviolet radiation, we have included this notation the first time quoted, see page 4 line 140, thanks for the remark.

Please include additional information regarding pomegranate and cocoa extracts, such as: product name and number, lot/batch number, full ingredient list, method of chemical characterization, purity and yield. Please also include a description of the look and feel of the substance, and the species name of the cocoa plant used.

We added the following paragraph: "The extracts were supplied by Naturex S.A. (Avignon, France). Pomegranate extract (reference EA140914, batch no D047/002/A11) was obtained from Punica granatum L. rinds (plant extract ratio between 5:1 and 7:1). The extraction solvent contains 70% ethanol, 30% water. The nutritional composition of the extract was as follows: energy 367 kcal/100 g; carbohydrates 84% (w/v); fat 1.4% (w/v); fiber 3.8% (w/v); proteins 2.4% (w/v), and total specific phenolic 12.62% (w/v)with, respectively, 2.77%; 7.92%, and 1.93% (w/v) ofpunicalagin A, punicaligin B, and ellagic acid. Cocoa extract (batch no TCP 19013) was obtained from Theobroma cacao L. complete seeds with the method patented by the company (EP2071961A1). The resulting product is a powdered semi-purified extract with a total polyphenol content of 20–45% and an organic fraction, from which, after concentration and drying thereof, a powdered purified extract is obtained with a total polyphenol content of 60–90%. The content of flavonols flavanol monomers catechin (24.2) and epicatechin (115) and 226.6 of total oligomeric procyanidins, flavonols (quercetin-3-O-glucoside 1.0; quercetin-3-O-arabinoside 1.0), anthocyanins and alkaloids (10.3 theobromine and 1.1 caffeine). "

PLOS requires an ORCID iD for the corresponding author in Editorial Manager on papers submitted after December 6th, 2016. Please ensure that you have an ORCID iD and that it is validated in Editorial Manager. To do this, go to 'Update my Information" (in the upper left-hand corner of the main

menu), and click on the Fetch/Validate link next to the ORCID field. This will take you to the ORCID site and allow you to create a new iD or authenticate a pre-existing iD in Editorial Manager. Please see the following video for instructions on linking an ORCID iD to your Editorial Manager account: https://www.youtube.com/watch?v=_xcclfuvtxQ

We have added at the metadata the ORCID iD of the corresponding author of the present submission.

Please ensure that you refer to Figures 6 and 7 in your text as, if accepted, production will need this reference to link the reader to the figure.

Both figures has been quoted in the text.

Please include a separate title for each table in your manuscript. The two tables are labeled as figures and the title of them is stated at the caption.

<u>Answers to remarks from referees</u>

<u>Referee 1</u> **In this study, the authors have examined the effect of pomegranate and cocoa extracts on UVA-induced skin cancer in SKH-1 hairless mice. Overall, whereas the studies are conducted properly, the model itself has serious concerns. Specifically, UVA radiation also does not induce skin cancers, and indeed the authors did not find any skin cancer in control group; except some kind of invasion which is not that common for non-melanoma skin cancer. Because of the same issue of using UVA, the effects of the test agents are also not evident, though they have shown efficacy against UVB-induced skin cancer; therefore, the studies lack both the novelty and appropriate model. Lastly, the molecules analyzed at the the study end are also not properly justified, and in most cases there is hardly any effect.**

As we say in the manuscript, in the eighties it was considered that while UVB played a key role in initiating tumors, UVA energy just intervened in promoting it (Forbes et al., see [11] in our manuscript). But the current consensus is that although UVB is approximately 1.000-10.000 times more carcinogenic UVA, chronic UVA radiation alone is also carcinogenic as shown in diverse in vivo experiments since 1990's (De Laat, Van Der Leun, and De Gruijl, 1997, see [20] in our manuscript). In recent studies, UVA rays has been confirmed to reach the deep dermis, and generating reactive oxygen species (ROS) which lead to oxidative

stress causing DNA damage similar to UVB and DNA strand breaks (Schuch, 2017). If the dose of UV exceeds a damage response threshold, keratinocytes activate apoptotic pathways (Bayerl, 1995). UVA causes also the degradation of extracellular matrix of the dermis by activation of matrix metalloproteinases (MMP) and the inhibition of collagen synthesis. UVA radiation also induces DNA strand breaks and DNA-protein cross-links (De Grujil, 2000, see [14] in our manuscript). UVB, is a potent stimulator of inflammation and the formation of DNA photolesions (such as mutagenic thymine dimers), whereas UVA is much less active in these measures but instead is a potent driver of oxidative free radical damage to DNA and other macromolecules. Thus, each may contribute to carcinogenesis through different mechanisms. But both UVA and UVB are responsible for pigmentation, photoaging, photoimmunosuppression and photocarcinogenesis (D'Orazio et al, 2013; Abadie et al, 2019). As we could confirm in our research, with the radiation source used, a 100% incidence of spinocellular carcinomas in the Control Group was observed. Finally we would like to highlight that to the WHO, both UV-A and UV-B contribute to DNA damage, and classified the use of UV-emitting tanning devices (sunbeds) as carcinogenic to humans. Artificial tanning devices: public health interventions to manage sunbeds, World Health Organization 2017, ISBN 978-92-4-151259-6.

Schuch, A.P.; Moreno, N.C.; Schuch, N.J.; Menck, C.F.M.; Garcia, C.C.M. Sunlight damage to cellular DNA: Focus on oxidatively generated lesions. Free Radic. Biol. Med., 2017, 107, 110–124

Bayerl, C.; Taake, S.; Moll, I.; Jung, E.G. Characterization of sunburn cells after exposure to ultraviolet light. Photodermatol. Photoimmunol. Photomed. 1995, 11, 149–154

Abadie, S., Bedos, P., & Rouquette, J. (2019). A human skin model to evaluate the protective effect of compounds against UVA damage. International Journal of Cosmetic Science. doi:10.1111/ics.12579

D'Orazio J, Jarrett S, Amaro-Ortiz A, Scott T. UV radiation and the skin. Int J Mol Sci. 2013; 14(6):12222-48. doi: 10.3390/ijms140612222.

Nevertheless, it is true that, as tumor genesis is faster with UVB, most research into UV-related pathogenesis and particularly those works investigating preventative or palliative treatment have used UVB radiation sources (Kim et al., 2016 see [19] in our manuscript; Rigby et al., 2017 see [25] in our manuscript; Afaq et al., 2010 see [1] in our manuscript; Park et al., 2010 see [23] in our manuscript). That is the reason why we

decided to use a source of radiation that emits mostly UVA, avoiding reproducing studies previously performed. As we said before, UVA damage is mediated by ROS generation. ROS such as hydrogen peroxides and hydroxyl radicals are important in the induction of mutations by UVA radiation. These ROS are generated by an endogenous photosensitizer (not DNA but, for example, porphirines or NADH) that becomes excited by absorbing UVA radiation, and then reacts with oxygen (a "type II reaction"; in a "type I reaction" the sensitizer reacts directly with the target molecule, e.g., DNA). Singlet oxygen can also be generated by UVA radiation through this sensitizing mechanism (De Grujil, 2000). Due to this mechanism of action, our research hypothesis stated that the use of two powerful antioxidants (widely tested) (Davinelli, 2018; Mogollon, 2014; Bassiri-Jahromi, 2018) could reverse the damage caused by UVA radiation, as we think has happened.

De Gruijl, F.R. Photocarcinogenesis: UVA vs. UVB. Methods Enzymol. 2000, 319, 359–366

Davinelli S, Corbi G, Zarrelli A, Arisi M, Calzavara-Pinton P, Grassi D, De Vivo I, Scapagnini G. Short-term supplementation with flavanol-rich cocoa improves lipid profile, antioxidant status and positively influences the AA/EPA ratio in healthy subjects.J Nutr Biochem. 2018 Nov;61:33-39. doi: 10.1016/j.jnutbio.2018.07.011. Epub 2018 Aug 14.

Mogollon JA, Boivin C, Lemieux S, Blanchet C, Claveau J, Dodin S. Chocolate flavanols and skin photoprotection: a parallel, double-blind, randomized clinical trial. J Nutr Health Nutr J. 2014 Jun 27;13:66. doi: 10.1186/1475-2891-13-66.

Bassiri-Jahromi S. Punica granatum (Pomegranate) activity in health promotion and cancer prevention. Oncol Rev. 2018 Jan 30;12(1):345. doi: 10.4081/oncol.2018.345

Referee 2 **This is an excellent article that investigates the protective effects of pomegranate and cocoa extracts during UVA-induced skin cancer in SKH-1 hairless mice. In general, it is very well written. The research question is clear. Robust, well-explored and well-described methods were used. Authors demonstrated that 100% of control animals presented spinocellular carcinomas which highlights the fact that UVA radiation can provoke the entire spectrum of photoaging lesions. Animals that received purified extracts of cocoa and pomegranate showed significant reductions in the incidence of skin carcinoma in both groups. In addition, lower mutated p53 expression**

in the treatment groups than in the control as well as, control group demonstrated higher PCNA and MMP-9 labelling. The present study is a step toward better understanding the UVA photocarcinogenesis and new chemopreventive strategies. However, some changes in the article are needed. I have the following suggestion and criticism. - Please add the reference of GLOBOCAN report. Check the incidence of skin cancer reported on it. Done. **Separate the introduction into three paragraphs to make it easier to follow. 2nd- Punica granatum... 3rd- SKH1/CRL...** Done.

- Materials and methods UV irradiation- Please inform the reference for the protocol used. - At the end of the experiment, animals were sacrificed by CO2 overdose. Why did you use CO2 chamber? Nowadays, it is recommended euthanasia using an isoflurane anesthetic overdose

According to the regulations in force in Spain, rodents can be slaughtered with the gradual release of CO2 (RD 53/2013) and is the method commonly used in the university stall, where the study has been conducted.

Results- Please change this title for Results and Discussion Done.

Please separate the following information in new paragaraph- " The protein p53 has diverse and complex 227 functions, including cell cycle regulation. Its alteration is directly related to cancer 228 development, as it mutates in 50% of human cancers, including NMSC (Benjamin and 229 Ananthaswamy, 2007) [5]. In SKH-1 mice chronically exposed to UVB radiation, 230 inactivation of p53 mutations has been reported to occur in 50-70% of tumors, this 231 being an early event, detected as early as 1 week post-UVB exposure (Rigby et al. 232 2017) [25]. The present study found lower mutated p53 expression in the treatment 233 groups than in the control, with statistically significant differences, but without any 234 significant difference between PGE and CE (Figures 3 and 5). After UVB exposure, 235 oral treatment with PGE, has also shown a positive effect on the expression of tumor 236 suppressor p53 and cyclin kinase inhibitor p21(Afaq et al., 2010) [1]." Done.

"But TIMP-1 showed more expression in the treatment groups, and so it would appear that inhibition of ECM degradation was

more active in these groups, although differences did not reach statistical significance (Figure 2, Figure 7)." If you did not have statistical differences the groups are equals. Rewrite the sentence!

We have rewritten the sentence in the following way:

"But TIMP-1 showed bigger value in the treatment groups, and so it would appear that inhibition of ECM degradation tended to be more active in these groups, although without reaching statistical significance differences (Figure 2, Figure 7)"

Referee 3 **The paper with the title Chemopreventive effect of pomegranate and cocoa extracts on ultraviolet radiation-induced photocarcinogenesis in SKH-1 mice is well write and the study design is conducted correctly. For these reasons the paper is accepted for the publication on PLOS ONE.**

Thanks for your comment.
